# Cancer screening activity results in overdiagnosis and overtreatment of papillary thyroid cancer: A 10-year experience at a single institution

**DOI:** 10.1371/journal.pone.0236257

**Published:** 2020-07-21

**Authors:** Krzysztof Kaliszewski, Dorota Diakowska, Beata Wojtczak, Jerzy Rudnicki

**Affiliations:** 1 Department of General, Minimally Invasive and Endocrine Surgery, Wroclaw Medical University, Wroclaw, Poland; 2 Department of Nervous System Diseases, Faculty of Health Science, Wroclaw Medical University, Wroclaw, Poland; Medical Faculty of Porto University and IPATIMUP, PORTUGAL

## Abstract

**Background:**

It is estimated that one of the potential cause of the increasing prevalence of thyroid cancer (TC) is the easier and widespread access to diagnostic tools. If an individual evaluates the thyroid gland due to a mentioned mechanism without considering TC risk factors or symptoms, we can describe this phenomenon as cancer screening activity (CSA).

**Aim of the study:**

We 1) estimated what types of TC were diagnosed due to CSA, 2) analyzed what clinicopathological features were characteristic of TCs diagnosed by CSA, 3) determined if these features were characteristic of indolent cases, and finally we 4) assessed whether CSA could have resulted in the increasing incidence of potentially indolent papillary thyroid cancer (PTC).

**Materials and methods:**

A retrospective review of 4,701 medical records of patients admitted and surgically treated at one surgical center between 2008 and 2017 was performed. Among the enrolled patients, 569 (12.1%) had thyroid malignancy, and 514 (10.9%) were diagnosed with PTC. We divided these patients into two groups: 1) patients in whom TC diagnostics were performed without considering any TC risk factors or symptoms (CSA-yes) and 2) those in whom TC was diagnosed due to TC risk factors or symptoms (CSA-no). We then compared the clinicopathological features of these two groups.

**Results:**

The most common type of TC diagnosed in the CSA-group was PTC (p = 0.024). CSA-yes patients showed a significantly lower degree of Tumor-Node-Metastasis (TNM) staging and demonstrated a significantly lower rate of multifocality, but not of bilaterality (p<0.0001 and p = 0.198, respectively). In the CSA-yes group, the number of TC foci was significantly lower than that in the CSA-no group (p<0.0001). All clinicopathological features characteristic of aggressive cases of TC were absent in CSA-yes patients (p<0.0001), while all features observed in CSA-yes patients were characteristic of indolent cases (p<0.0001).

**Conclusions:**

The use of CSA results in the diagnosis of indolent cases of PTC and may be one of the potential causes of overdiagnosis and overtreatment of this malignancy.

## Introduction

In managing papillary thyroid cancer (PTC), there exists a basic question: where is the border between evidence-based surgical management and overtreatment [1] Additionally, this dilemma is also emphasized due to potential complications, which may appear after the surgical procedure [2]. Perhaps if we could cure patients of thyroid cancer (TC) without any risk of iatrogenic injuries, this question might not be considered highly.

What exactly do we mean by overdiagnosis and overtreatment of PTC? In the case of this specific malignancy, overdiagnosis and overtreatment are observed when the tumor is potentially diagnosed and treated correctly but may result in an unfavorable balance between benefits and adverse events [3, 4]. Specifically, in PTC management, overdiagnosed tumors are those that did not require management or treatment because they would not have produced any symptoms or even led to death [5].

Because of the increasing prevalence of PTC diagnosis but not its mortality in recent decades [1, 5], we have focused our concerns on what is responsible for this epidemiological situation.

Based on previous experience with other neoplasms in which a screening program was introduced, a rapidly increasing rate of their prevalence without a concomitant increase in their mortality is observed [6]. Such a situation exists in the case of a prostate cancer screening program [6]; however, does PTC suffer from the same issue?

To date, there are no registered TC screening systems in Poland or even in Europe. However, what would be the value of a potential TC screening program? Would it only discovered TC cases that are slow-growing and indolent or are the least likely to harm anyone? Would the program have a lower ability to diagnose aggressive, fast-growing tumors, which cause TC mortality? The answer to these questions may be that in the case of PTC management, a screening program would cause overdiagnosis and overtreatment. However, omitting the overall statistical results [7], it is extremely difficult to judge whether an individual diagnosed with papillary thyroid microcarcinoma (PTMC) falls under the category of overdiagnosis [8]. Of course, we are aware of the potential excellent prognosis of PTC; however, in some cases, highly aggressive entities might be observed [9]. If the patient undergoes surgical treatment, we would not be able to determine what would have happened in the absence of surgery. Overdiagnosis and overtreatment of PTC can be measured or statistically assessed only in a large, untreated, prospective, randomized group. Thus, in diagnosing PTC, should everyone be treated individually? Therefore, the next argument addresses the overdiagnosis and overtreatment of PTC.

In the current epidemiological circumstances, it is estimated that the increasing prevalence of PTC is also caused by easier and widespread access to diagnostic tools [1, 3–6]. When TC is diagnosed based on an established mechanism without considering TC risk factors or symptoms, we describe this phenomenon as cancer screening activity (CSA). By contrast, can we subsequently say that this unofficial screening program achieves the fundamental goals for a cancer screening system, i.e., reducing suffering and the number of deaths caused by TC, without introducing any significant additional harm? As difficult as this question is, we decided to give the best answer possible.

Here, we evaluated the fundamental topics concerning PTC management. First, we discussed the main potential reasons for CSA for TC and the increasing prevalence of this malignancy. Second, we estimated what types of TCs were discovered due to CSA. Third, we determined what clinicopathological features were characteristic of TCs diagnosed by CSA and whether these features were characteristic of indolent or aggressive cases. Finally, we assessed whether the behavior (CSA) of the diagnostician might have resulted in overdiagnosis and overtreatment of TC.

## Materials and methods

Our study protocol was approved by the Bioethics Committee of Wroclaw Medical University, Poland (Signature number: KB-783/2017). We obtained verbal consent from the participants instead of written consent, because the data were retrospectively and anonymously analyzed from established medical records. Every verbal consent was witnessed by responsible physician and documented in medical history. The authors did not have access to identifying patient information or direct access to the study participants.

We performed retrospective chart reviews of 4,701 patients who were admitted and surgically treated due to thyroid tumors at a single medical center between 2008 and 2017. 20017–2019 was the date upon which this clinical data was accessed. Thyroid malignancy was coded according to the 3^rd^ edition of the International Classification of Diseases for Oncology (ICD-O-3). For our medical database, we used primary invasive TC cases (ICD-O-3: C73) recorded in our Hospital’s Center Cancer Registry (HCCR) between 2008 and 2017. TCs were staged in accordance with the Tumor-Node-Metastasis (TNM) staging criteria (Table 1) proposed by the AJCC 8^th^ Edition [10]. Preoperative thyroid ultrasonography, ultrasound-guided fine needle aspiration biopsy (UG-FNAB) and cytological examinations were performed in all cases. After surgery, the final histopathological classification was stated according to World Health Organization guidelines [11]. The excised tissue specimens were fixed in 10% buffered formalin and histopathologically diagnosed at the Department of Pathomorphology, Wroclaw Medical University, Poland. Representative blocks were selected. Because PTC can be multifocal, the adjacent and contralateral lobes were sampled, and all pale areas were processed. Serial sectioning of the representative tissue samples was carefully performed. The specimens were routinely process as follows. Sections were cut into 4-μm-thick slices, which were subjected to conventional hematoxylin and eosin (H&E) staining. H&E-stained sections were evaluated by two pathologists experienced in evaluating thyroid lesions to confirm the diagnosis, pathological features of the tumor and extent of the malignancy. In individuals younger than 55 years of age, stage I tumors were defined as early carcinoma, and stage II tumors were defined as advanced carcinoma. In individuals aged 55 or older, stage I (T1N0M0) and stage II tumors (T2N0M0) were defined as early carcinoma, whereas stage III (T3N0M0, T1-3N1aM0) and stage IV (all undifferentiated carcinomas) tumors were defined as advanced carcinoma [11]. We performed the analysis according to recent oncology guidelines using the European Society for Medical Oncology Classification (ESMO; www.esmo.org), National Comprehensive Cancer Network (NCCN; www.nccn.org) and American Thyroid Association (ATA) guidelines [11].

**Table 1:** The demographic, clinical and histopathological characteristics of thyroid cancer patients divided into the following two groups according to the implementation of cancer screening activity (CSA): CSA-yes and CSA-no. Descriptive data are presented as a number (percent) or mean ±SD.

Variable	CSA-yes (n-248)	CSA-no (n = 321)	P-value
Gender:			0.974
Male	35 (14.1)	45 (14.0)	
Female	213 (85.9)	276 (86.0)	
Age (years)	49.6 ± 15.9	52.0 ± 14.7	0.059
Age:			0.453
<55	143 (57.7)	175 (54.5)	
≥55	105 (42.3)	146 (45.5)	
Histopathological type:			<0.0001
Papillary	244 (98.4)	263 (81.9)	
Follicular	1 (0.4)	19 (5.9)	
Medullary	3 (1.2)	16 (5.0)	
Undifferentiated	0 (0.0)	11 (3.4)	
Sarcoma	0 (0.0)	3 (0.9)	
Secondary	0 (0.0)	3 (0.9)	
Squamous	0 (0.0)	2 (0.6)	
Lymphoma	0 (0.0)	3 (0.9)	
Plasmocytoma	0 (0.0)	1 (0.3)	
pTNM stage:			<0.0001
I	244 (98.4)	194 (60.4)	
II	2 (0.8)	73 (22.7)	
III	2 (0.8)	26 (8.1)	
IV	0 (0.0)	28 (8.7)	
pT:			<0.0001
T1a	107 (43.2)	96 (29.9)	
T1b	137 (55.2)	117 (36.5)	
T2	3 (1.2)	65 (20.2)	
T3	1 (0.4)	15 (4.7)	
T4a	0 (0.0)	11 (3.4)	
T4b	0 (0.0)	17 (5.3)	
pN:			<0.0001
N0	198 (79.8)	134 (41.7)	
N1a	33 (13.3)	114 (35.5)	
N1b	1 (0.4)	16 (5.0)	
Nx	16 (6.5)	57 (17.8)	
pM:			<0.0001
M0	241 (97.2)	242 (75.4)	
M1	1 (0.4)	13 (4.0)	
Mx	6 (2.4)	66 (20.6)	
Minimum cN1a:			<0.0001
Yes	30 (12.1)	110 (34.3)	
No	218 (87.9)	211 (65.7)	
Type of tumor:			<0.0001
Solitary	225 (90.7)	179 (55.9)	
Multifocal	23 (9.3)	141 (44.1)	
Bilateral (n = 567):			0.200
Yes	14 (5.7)	27 (8.4)	
No	234 (94.4)	294 (91.6)	
Presence of TC symptoms:			<0.0001
Yes	0 (0.0)	194 (60.4)	
No	248 (100.0)	127 (39.6)	
Risk factors of TC:			<0.0001
Yes	0 (0.0)	53 (16.5)	
No	248 (100.0)	268 (83.5)	
Presurgical diagnosis of multifocality:			0.936
Yes	12 (4.8)	16 (5.0)	
No	236 (95.2)	305 (95.0)	
Presurgical diagnosis of bilaterality			0.469
Yes	4 (1.6)	8 (2.5)	
No	244 (98.4)	313 (97.5)	
Thyroid malignancy:			<0.0001
Before surgery	220 (88.7)	133 (41.4)	
After surgery	28 (11.3)	188 (58.6)	
Type of surgery:			<0.0001
Total	216 (87.1)	182 (56.7)	
No total	32 (12.9)	139 (43.3)	
Reoperation needed:			<0.0001
No	221 (89.1)	192 (59.8)	
Yes	27 (10.9)	129 (40.2)	
Number of foci (n = 559):			<0.0001
1	224 (90.3)	236 (73.5)	
2	12 (4.8)	76 (23.7)	
3	12 (4.8)	9 (2.8)	
Recurrence:			<0.0001
Yes	0 (0.0)	57 (17.8)	
No	248 (100.0)	264 (82.2)	
Deaths due to TC:			<0.0001
Yes	0 (0.0)	38 (11.8)	
No	248 (100.0)	283 (88.2)	

CSA: Cancer Screening Activity

**Fig 1:**
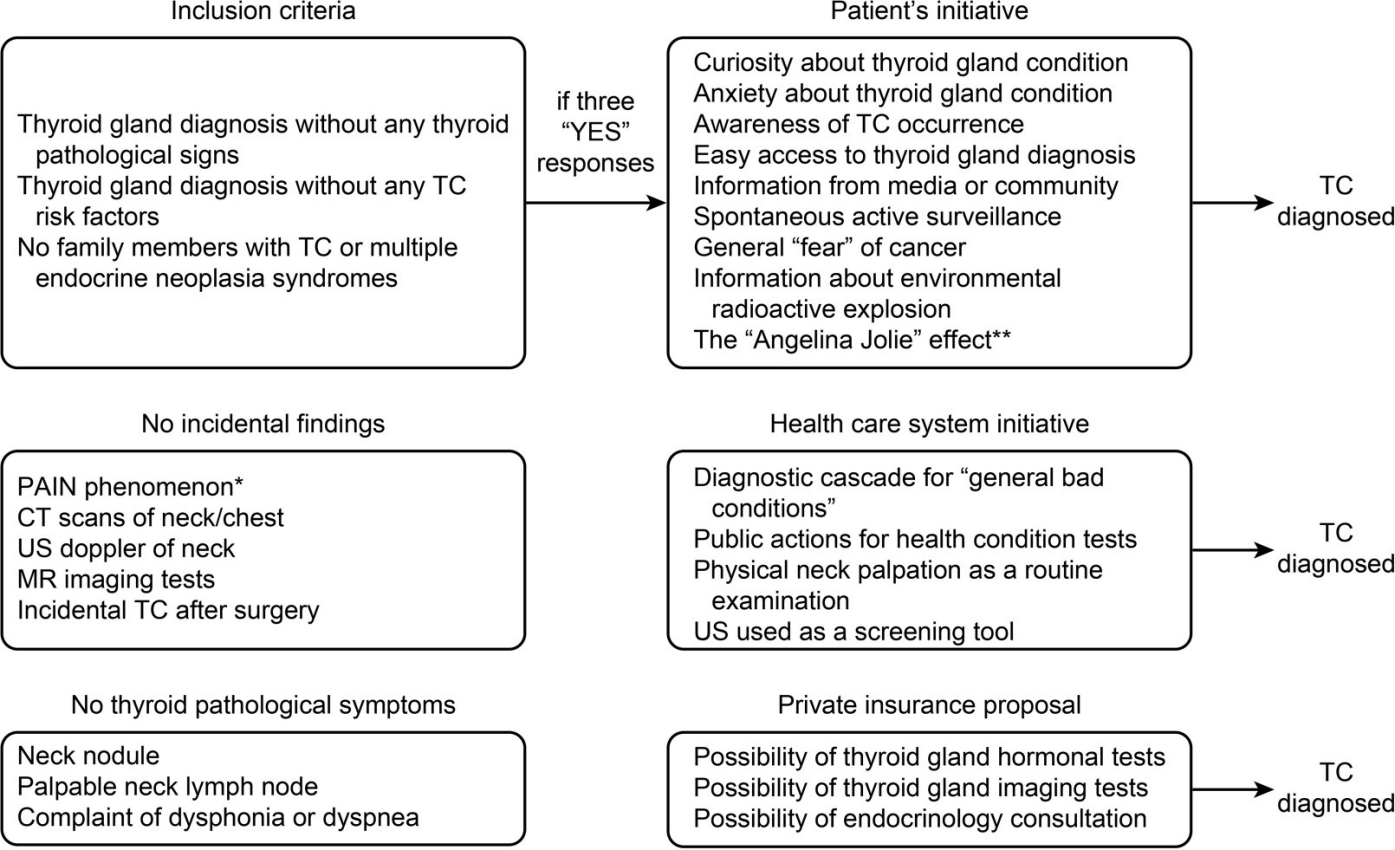
Cancer screening activity mechanism in patients with thyroid cancer diagnosed in 2008–2017. *[36] **[41].

Among the 4,701 patients, 569 (12.1%) had a thyroid malignancy, and 514 (10.9%) were diagnosed with PTC. All histopathological types of TC diagnosed in 2008–2017 are presented in Table 2. Next, using the medical records of all patients with TC, we extracted these data, which conveyed a diagnosis of TC after meeting the CSA criteria (Fig 1).

**Table 2:** Comparison of the histopathological types of thyroid cancer and cancer screening activity in thyroid cancer patients from 2008–2017. Descriptive data are presented as numbers (percentages).

Histopathological type of TC	2008	2009	2010	2011	2012	2013	2014	2015	2016	2017	Total (n = 569)
PTC:											N = 507:
CSA-yes	8 (28.6)	9 (23.1)	10 (25.0)	10 (31.3)	15 (25.0)	23 (36.5)	20 (55.6)	54 (66.7)	51 (69.9)	44 (80.0)	244 (48.1)
CSA-no	20 (71.4)	30 (76.9)	30 (75.0)	22 (68.7)	45 (75.0)	40 (63.5)	16 (44.4)	27 (33.3)	22 (30.1)	11 (20.0)	263 (51.9)
FTC:											N = 20:
CSA-yes	0 (0.0)	0 (0.0)	0 (0.0)	0 (0.0)	0 (0.0)	0 (0.0)	0 (0.0)	1 (20.0)	0 (0.0)	0 (0.0)	1 (5.0)
CSA-no	1 (100.0)	0 (0.0)	2 (100.0)	3 (100.0)	4 (100.0)	3 (100.0)	2 (100.0)	4 (80.0)	0 (0.0)	0 (0.0)	19 (95.0)
MTC:											N = 19:
CSA-yes	0 (0.0)	0 (0.0)	0 (0.0)	2 (100.0)	0 (0.0)	0 (0.0)	0 (0.0)	1 (14.3)	0 (0.0)	0 (0.0)	3 (15.8)
CSA-no	1 (100.0)	0 (0.0)	2 (100.0)	0 (0.0)	1 (100.0)	0 (0.0)	2 (100.0)	6 (85.7)	3 (100.0)	1 (100.0)	16 (84.2)
UTC:											N = 11:
CSA-yes	0 (0.0)	0 (0.0)	0 (0.0)	0 (0.0)	0 (0.0)	0 (0.0)	0 (0.0)	0 (0.0)	0 (0.0)	0 (0.0)	0 (0.0)
CSA-no	2 (100.0)	0 (0.0)	4 (100.0)	1 (100.0)	1 (100.0)	1 (100.0)	0 (0.0)	0 (0.0)	1 (100.0)	1 (100.0)	11 (100.0)
Sarcoma:											N = 3:
CSA-yes	0 (0.0)	0 (0.0)	0 (0.0)	0 (0.0)	0 (0.0)	0 (0.0)	0 (0.0)	0 (0.0)	0 (0.0)	0 (0.0)	0 (0.0)
CSA-no	1 (100.0)	0 (0.0)	0 (0.0)	0 (0.0)	1 (100.0)	0 (0.0)	1 (100.0)	0 (0.0)	0 (0.0)	0 (0.0)	3 (100.0)
Secondary:											N = 3:
CSA-yes	0 (0.0)	0 (0.0)	0 (0.0)	0 (0.0)	0 (0.0)	0 (0.0)	0 (0.0)	0 (0.0)	0 (0.0)	0 (0.0)	0 (0.0)
CSA-no	0 (0.0)	0 (0.0)	0 (0.0)	0 (0.0)	0 (0.0)	2 (100.0)	1 (100.0)	0 (0.0)	0 (0.0)	3 (100.0)	3 (100.0)
Squamous:											N = 2:
CSA-yes	0 (0.0)	0 (0.0)	0 (0.0)	0 (0.0)	0 (0.0)	0 (0.0)	0 (0.0)	0 (0.0)	0 (0.0)	0 (0.0)	0 (0.0)
CSA-no	0 (0.0)	0 (0.0)	0 (0.0)	0 (0.0)	0 (0.0)	0 (0.0)	0 (0.0)	0 (0.0)	0 (0.0)	2 (100.0)	2 (100.0)
Lymphoma:											N = 3:
CSA-yes	0 (0.0)	0 (0.0)	0 (0.0)	0 (0.0)	0 (0.0)	0 (0.0)	0 (0.0)	0 (0.0)	0 (0.0)	0 (0.0)	0 (0.0)
CSA-no	0 (0.0)	0 (0.0)	0 (0.0)	0 (0.0)	0 (0.0)	0 (0.0)	0 (0.0)	0 (0.0)	0 (0.0)	3 (100.0)	3 (100.0)
Plasmocytoma:											N = 1:
CSA-yes	0 (0.0)	0 (0.0)	0 (0.0)	0 (0.0)	0 (0.0)	0 (0.0)	0 (0.0)	0 (0.0)	0 (0.0)	0 (0.0)	0 (0.0)
CSA-no	0 (0.0)	0 (0.0)	0 (0.0)	0 (0.0)	0 (0.0)	0 (0.0)	0 (0.0)	0 (0.0)	0 (0.0)	1 (100.0)	1 (100.0)

PTC: papillary thyroid carcinoma; FTC: follicular thyroid carcinoma; MTC: medullary thyroid carcinoma; UTC: undifferentiated thyroid carcinoma; CSA: cancer screening activity

### Cancer screening activity (CSA) inclusion criteria

- *Initiation of thyroid gland diagnostics* *without any pathological signs of thyroid dysfunction*
- *Initiation of thyroid gland diagnostics* *in the absence of any TC risk factors or symptoms*
- *No family history of TC* *or multiple endocrine neoplasia syndrome*

In our study, we analyzed the cases that met all the criteria for CSA and additionally presented a significant increase. Patients who, on their own, decided to undergo TC diagnostics without any TC symptoms and without any TC risk factors were assigned to the CSA-yes group. Next, we compared this group with the patients in whom TC was diagnosed but only due to the presence of at least one TC clinical symptom or risk factor (CSA-no group). On the basis of a medical records review, we proposed the most common reasons for CSA (Fig 1).

### Reasons for engaging in cancer screening activity (CSA)

- *Easy access to thyroid* *gland diagnostics*
- *Curiosity/anxiety regarding* *thyroid gland condition*
- *Awareness of* *TC occurrence*
- *Private insurance’s* *proposal for thyroid gland examination*
- *Diagnostic cascade for “general bad conditions” i*.*e*. *malaise*, *dizziness*, *shortness of breath*, *choking*, *feeling of anatomical mass in the neck*.
- *Public actions for* *monitoring health conditions*

The CSA-yes group comprised 248 (5.27%) patients, whereas the CSA-no group comprised 321 (6.82%) patients (Table 1). There were 35 (14.2%) males and 212 (85.8%) females in the CSA-yes group and 45 (14.1%) males and 275 (85.9%) females in the CSA-no group (p = 0.971). Next, we analyzed the numbers of patients with TC in 2008–2017 who were diagnosed via CSA (CSA-yes) and traditional methods (CSA-no) (Table 2). To determine the diagnosis of TC with potentially aggressive or indolent behaviors, we compared TC clinical and histopathological features with the aggressiveness of the disease (8); features included echogenicity, tumor size at the time of diagnosis, microcalcifications, tumor shape, margins and vascularity. Finally, we determined how many TCs were diagnosed before and after surgery in the CSA-yes and CSA-no groups. We hypothesized that individuals in the CSA-yes group did not confirm the malignant nature of their tumors when they finally underwent surgery after TC diagnosis. We also investigated whether individuals in the CSA-yes group reported a higher number of presurgically diagnosed TCs and whether these numbers were lower or higher at the beginning and end of the time period. This observation additionally addressed the quality of presurgical diagnostics during the time period (2008–2017).

### Statistical analysis

Data were analyzed using Microsoft Excel 2016 and Statistica 13.3 (Tibco software Inc. CA, USA). Descriptive data are presented as the number of observations and percentages or as the mean and standard deviation (±SD). Qualitative variables were compared using chi-square and Fisher exact tests, and quantitative variables were analyzed by Student’s t test. P-values below 0.05 were considered statistically significant.

## Results

**Fig 2:**
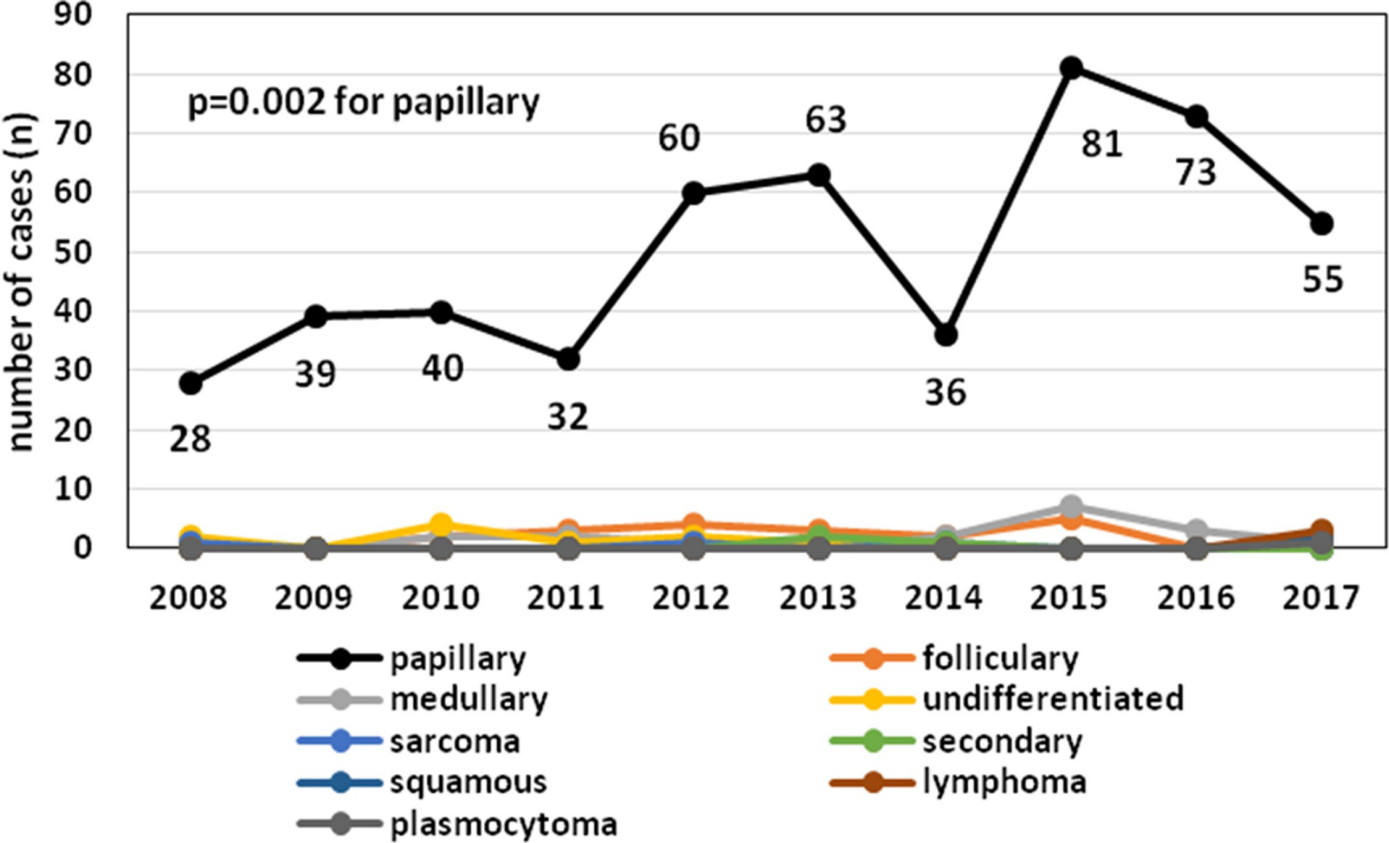
The number of patients with papillary thyroid cancer increased significantly compared to that of the patients with other types of thyroid cancer from 2008–2017 (p = 0.024).

**Fig 3:**
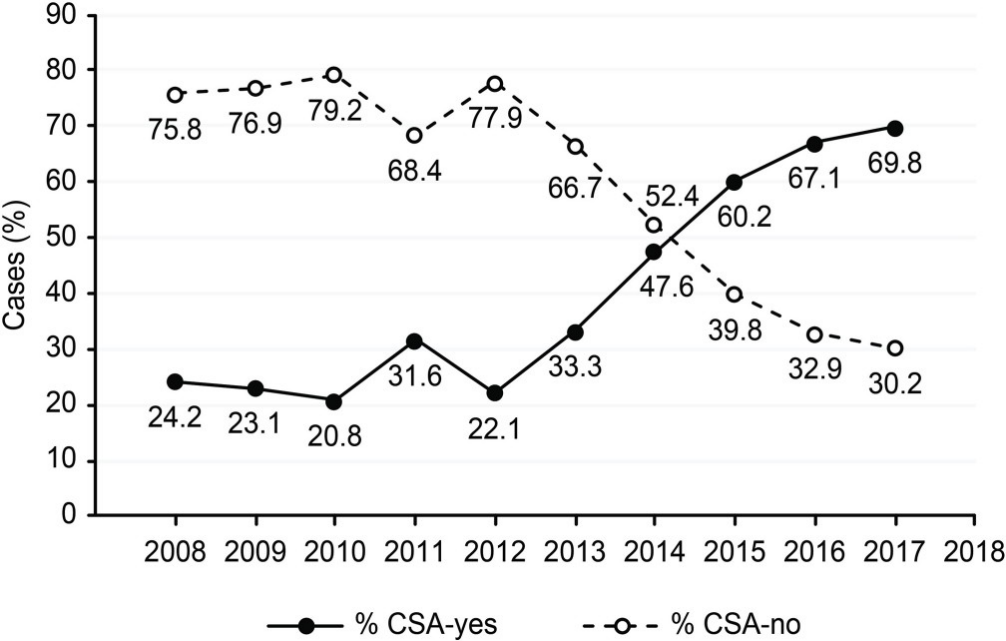
Percent of thyroid cancer patients in the CSA-yes and CSA-no groups from 2008–2017.

**Fig 4:**
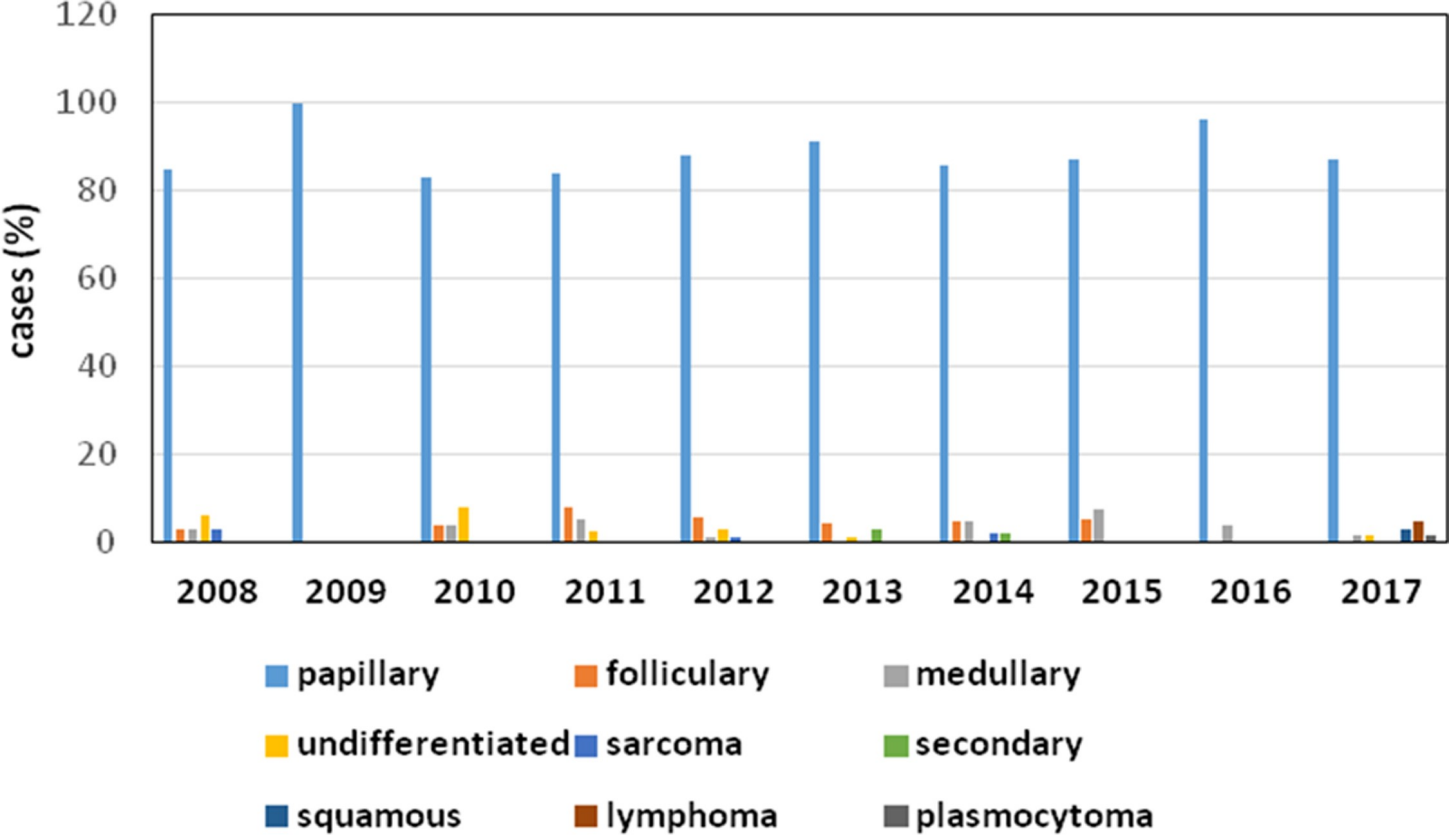
Percent of patients diagnosed with the histopathological types of thyroid cancer from 2008–2017.

**Fig 5:**
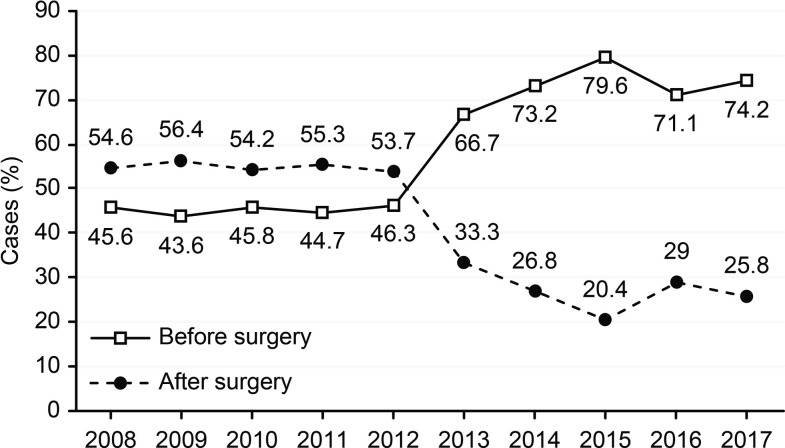
Percent of patients with a diagnosis of thyroid cancer before surgery and after surgery from 2008–2017.

The demographic characteristics of the CSA-yes (n = 248) and CSA-no (n = 321) groups are presented in Table 1. We did not observe a statistically significant difference in age between the two groups; however, there was a trend of younger patients (<55 years) in the CSA-yes group (p = 0.453). Patients with TC in the CSA-yes group showed a significantly lower degree of TNM staging (p<0.0001 for all features) (Table 1). In CSA-yes patients, we observed significantly lower rates of multifocality, but not bilaterality (p<0.0001 and p = 0.198, respectively), and a significantly lower number of TC foci than those in CSA-no patients (p<0.0001). We did not notice any instances of TC recurrence among the patients in the CSA-yes group but observed 57 cases of disease recurrence in the CSA-no group (p<0.0001). To date, none of the 248 patients in the “CSA-yes” group died due to TC. Based on questionnaire responses, we confirmed that 38 patients in the CSA-no group died of TC disease (p<0.0001), and the majority of deaths were caused by TC rather than PTC. As we show in Table 2, the increasing rate of TC in the analyzed period (2008–2017) concerned mainly PTC (Table 2 and Fig 2). In 2008, we observed 28 cases of PTC (8 were diagnosed due to CSA), while in 2015–2017, there were 81, 73 and 61 cases of PTC, respectively. We also noticed that the number of TCs diagnosed due to CSA during the analyzed period (2008–2017) increased from 24.2% in 2008 to 69.8% in 2017 (Fig 3). During this time, the most common type of TC was PTC (Fig 4). We observed 4 cases of TC excluding PTC diagnosed due to CSA (follicular thyroid cancer (FTC), 1 case and medullary thyroid cancer (MTC), 3 cases) (Table 2). All clinicopathological features that are characteristic of potentially aggressive TC behavior were not observed in patients in the CSA-yes group but were predominant in patients in the CSA-no group (p<0.0001 for all variables) (Table 3). In the CSA-yes group, we observed small, low-risk tumors (Table 3). Finally, we performed an analysis of TC presurgical diagnosis in the CSA-yes and CSA-no groups (CSA-yes vs. CSA-no) (Table 4) and estimated that the number of patients with TC diagnosed before surgery was lower in both groups at the beginning of the trial period and higher at the end of this period. We confirmed this increase and observed that it occurred in both groups (Fig 5).

**Table 3:** Selected ultrasound features of thyroid cancer patients divided into two groups according cancer screening activity (CSA): CSA-yes and CSA-no. Descriptive data are presented as numbers (percent).

Variable	CSA-yes (n-248)	CSA-no (n = 321)	P-value
Hypoechogenicity:			<0.0001
Yes	185 (74.6)	288 (89.7)	
No	63 (25.4)	33 (10.3)	
Tumor size:			<0.0001
<5 mm	54 (21.8)	27 (8.4)	
≥5 mm	194 (78.2)	294 (91.6)	
Microcalcifications:			<0.0001
Yes	37 (14.9)	281 (87.5)	
No	211 (85.1)	40 (12.5)	
Tumor shape:			<0.0001
Regular	210 (84.7)	42 (13.1)	
Irregular	38 (15.3)	279 (86.9)	
Sharpen margins:			<0.0001
Yes	213 (85.9)	34 (10.6)	
No	35 (14.1)	287 (89.4)	
Vascularity:			<0.0001
High	38 (15.3)	280 (87.2)	
Low	210 (84.7)	41 (12.8)	

CSA: Cancer Screening Activity

**Table 4:** Comparison of thyroid cancer diagnosis (before surgery vs after surgery) and cancer screening activity in TC patients from 2008–2017. Descriptive data are presented as numbers (percentages).

Thyroid malignancy	2008	2009	2010	2011	2012	2013	2014	2015	2016	2017	Total (n = 569)
Before surgery:											N = 353:
CSA-yes	7 (46.7)	8 (47.1)	10 (45.5)	8 (47.1)	12 (37.5)	22 (47.8)	17 (56.7)	52 (70.3)	51 (94.4)	33 (71.7)	220 (62.3)
CSA-no	8 (53.3)	9 (52.9)	12 (54.5)	9 (52.9)	20 (62.5)	24 (52.2)	13 (43.3)	22 (29.7)	3 (5.6)	13 (28.3)	133 (37.7)
After surgery:											N = 216:
CSA-yes	1 (5.6)	1 (4.5)	0 (0.0)	4 (19.0)	3 (8.3)	1 (4.3)	3 (25.0)	4 (21.1)	0 (0.0)	11 (64.7)	28 (13.0)
CSA-no	17 (94.4)	21 (95.5)	26 (100.0)	17 (81.0)	33 (91.7)	22 (95.7)	9 (75.0)	15 (78.9)	22 (100.0)	6 (35.3)	188 (87.0)

CSA: Cancer Screening Activity, TC: thyroid cancer

## Discussion

Our analysis showed that the majority of patients with TC were diagnosed without presenting any clinical symptoms, and all of them underwent an ultrasound test and subsequent UG-FNAB without showing any physical signs of TC. If these patients decided to have their thyroid gland examined in the absence of any physical symptoms of thyroid pathology, we described these behaviors as CSA. Every procedure was performed almost as in the unofficial cancer screening program, which has examined a large population of relatively healthy people. The tests that were used to identify these tumors were simple, cheap and easy to access. Despite the fact that CSA may result in overdiagnosis and overtreatment, we admit that these early-stage tumors were easier to treat and had excellent postsurgical prognosis. On this occasion, we emphasize that although the use of CSA might be questionable for managing PTC, for MTC CSA could result in its early diagnosis and subsequent treatment, leading to more favorable outcomes. Although we confirmed this observation in our study, further analyses are needed.

Regarding PTC management, there are some doubts about the ultimate goal of this unofficial cancer screening system, i.e., reducing suffering and death. In the CSA-yes group, we did not observe TC-related deaths, while in the CSA-no group, we observed 38 (11.8%) deaths due to TC. It is estimated, that overdiagnosis can result in harm [5], so we discourage the use of CSA for managing PTC because its leads to overtreatment. However, if PTC screening activity causes overdiagnosis and overtreatment, we can say that it damages the positive link between the purpose of screening systems and their ultimate goals. Therefore, in the end, if screening activity for PTC produces high rates of overdiagnosis and overtreatment, it is more likely to increase than decrease potential harm to patients. The main issue of PTC screening activity is its long-term effects. In our study, we obtained unquestionable results showing that almost all patients with TC discovered due to CSA had PTC without features associated with aggressive behaviors. Rather, they very often had small tumors (<5 mm), no microcalcifications, regular shapes, well-defined margins and low vascularity. Therefore, if CSA discovers only slow-growing, less aggressive PTC lesions, all diagnostic procedures leading to PTC findings might be unreliable. It may also lead to observations, that these specific PTC entities are asymptomatic for a very long time, so they are more likely to be caught by screening activity. On the other hand, aggressive and fast-growing entities manifest symptoms more quickly and force the patient to see a physician immediately. Therefore, there is the hypothesis that individuals with PTC who require surgery are not discovered by CSA but, on the contrary, during a standard physical examination between scheduled cancer screening visits [12]. This situation demonstrates an incredibly important feature of PTC: although TC screening activity appears to catch only dormant, indolent tumors, highly aggressive entities exist within this type of malignancy [9]. They may grow so quickly that even frequent screenings may not be able to catch them during the early stage. In our study, we estimated, that no patients with PTC diagnosed by CSA died. By contrast, in the CSA-no group, we observed 38 deaths; however, 32 of them were not diagnosed with PTC, but rather had more aggressive TC histological types. When analyzing all the facts, there is no clear definition of how tumors are overdiagnosed. If we want to confirm their overdiagnosis, we should follow such patients without any treatment unless they die of other causes unrelated to TC. Of course, we can estimate the overdiagnosis indirectly when we observe an increasing rate of TC but without concomitant increase in mortality [4]. The second indirect evidence of TC overdiagnosis and subsequent overtreatment might be the fact that we observed only an increase in the rate of early-stage TC diagnosis but not of advanced tumor diagnosis. Indeed, in our analysis, we estimated a large rise in the prevalence of early-stage PTC, such as PTMC [13]. We did not observe a similar situation in the incidence of the other types of TC. These observations support the hypothesis regarding the overdiagnosis and overtreatment of PTC. There are many studies that confirm a rapidly increasing rate of PTC with a slight decrease in mortality in high-income countries [14, 15]. In our analysis, we observed a growing proportion of operations performed due to TC, especially for PTC; this was also observed by other authors several years before our analysis [16]. They reported that the rate of thyroidectomies performed due to TC increased by 44% from 1999 to 2009. Some other researchers estimated that the increasing rate of thyroidectomies performed due to some other indications, such as goiter, were correlated with low-risk TC detection [17]. We described the same situation on the basis of our own observation study [18].

In some European countries such as France or England and in the United States, 45 to 70% of TCs were considered overdiagnosed [19] based on studies comparing the expected and observed TC prevalence. However, the increasing rate of TC prevalence in some other countries might be attributed to slightly different reasons. Since 1999, TC screening in South Korea has been commonly performed by general practitioners with ultrasound tests as a part of its National Cancer Control Program [20], what makes this country the place with the highest incidence of TC in the world. Interestingly, however, when in 2014 ultrasound screening in South Korea was discouraged, the incidence of TC and the number of its operations markedly decreased then [21]. Of course, we do not observe this phenomenon in Europe and in the United States, where the number of total thyroidectomies or hemithyroidectomies due to TC diagnosis has still increased [22, 23]. However, in some other previous analyses of the overdiagnosis and overtreatment of PTC, the authors go even further. Some have stated that the cancer screening program, as an unquestionable source of overdiagnosis and overtreatment, discovers tumors that may not progress or may even regress [24]. Nevertheless, according to the accessible literature concerning PTC observation, there are no reports describing such observations worldwide. However, Brawley stated that in some types of malignant tumors, specific lesions that become cancerous are only observed under the microscope because they are clinically silent [25]. This opinion was based of breast cancer observations, but we think that this view fits better with PTC behavior.

The next argument for the overdiagnosis and overtreatment of PTC is its very low recurrence rate after surgery [8]. In this study, we estimated that none of CSA-yes patients in whom PTC was diagnosed after surgery showed any signs of disease recurrence, while 17.8% of patients diagnosed with PTC based on clinical symptoms showed signs of TC recurrence. This is also why some authors contest the balance between the benefits and harms of cancer screening programs [26]. Esserman et al. [27] noticed that cancer screening programs produce more overdiagnosed TC entities, and we think that the same situation applies to CSA. Regarding PTC, some authors believe that the main contributor to the overdiagnosis and overtreatment of PTC is ultrasound of the thyroid and adjacent structures [6]. Others noticed that during recent decades, the prevalence of TC has markedly increased mainly in high-income countries [14]. Therefore, some authors predict that because TC has the fastest growing incidence among all malignant tumors, by 2030, it will be the fourth most common cancer in the world if observed trends are maintained [28]. However, in concordance with some other studies, we estimated that the increasing prevalence of TC is limited to PTC [29]. During our observation period, we noticed an increasing rate in only PTC, but not in FTC, MTC or undifferentiated TC.

The other issue regards the influence of the risk factors and increasing rate of PTC. We cannot exclude external risk factors for TC, the most commonly known of which is radioactive exposure; however, in Poland, we did not confirm the effects of this factor [30]. Although our country is located in Eastern Europe, it is difficult to assess the real impact on the rate of TC increase after the nuclear power plant accident in Chernobyl in 1986. However, after this disaster, there was an increase in the number of TC diagnoses in children and adolescents exposed to radioactive iodine in Belarus, Russia, and Ukraine [31]. Additionally, after the Fukushima Daiichi nuclear plant disaster in 2011, a large number of new TC diagnoses were estimated. However, at that time, TC screening was introduced [32]. Therefore, the question remains that if that increased rate was not the results of the screening itself, what was the cause?

The next estimated risk factor for TC is iodine deficiency. However, in 1996, under the auspices of the Ministry of Health, the Polish Council for the Control of Iodine Deficiency Disorders (PCCIDD) established a National Program for the Elimination of Iodine Deficiency Disorders to monitor iodine status and develop a mandatory model of iodine prophylaxis [33]. Therefore, because of a sufficient supply, this factor did not have any influence on the increasing rate of TC diagnosis observed in the study period. Other risk factors, such as hormone supplementation, obesity, diet, environment and genetics, have also been reported; however, there is no evidence to confirm their real impact on the increasing rate of TC diagnosis, but these factors obviously cannot be definitively excluded [34]. In the current study, we did not perform an analysis with these factors.

The next likely cause of the increased rate of detection of early-stage TC is advances in detection protocols. Easy access to high-frequency ultrasound neck examination or to the other imaging tests results in a high number of newly diagnosed cases of PTC. Many studies based on autopsy evaluations confirm the high number of undiagnosed PTCs [5, 35] and have highlighted three main estimated mechanisms that contribute to the increasing rate of newly diagnosed TC entities: opportunistic screening, i.e., palpation of asymptomatic patients with subclinical TC; diagnostic cascade performed in patients with general complaints, and incidental findings during neck imaging tests performed due to non-thyroid gland diseases [5, 36, 37]. In Poland, there are no recommendations for TC screening tests; however, ultrasound examinations of the thyroid and subsequent UG-FNAB are commonly ordered [38]. General practitioners’ easy access to some high-quality imaging tests in their practices may also be responsible for this situation. Additionally, in Poland, we also observed that easy access to healthcare, such as additional private insurance, increases the rate of new diagnoses of small PTC tumors [8].

As some authors state, CSAs such as registered, official cancer screening programs should be constantly monitored to be absolutely sure that screening for these specific malignancies provides more benefits than harm [39, 40]. Therefore, we think that assessments of protocols that result in overdiagnosis and overtreatment are urgently needed (Fig 5). Because of the harm potentially caused by overdiagnosis, the US Preventive Services Task Force has updated its recommendation to prevent routine screening activity for TC (D level recommendation) [5]. In addition, patients should be informed about their excellent prognosis, especially in cases of PTMC, and potential adverse events, which may manifest after surgical treatment [8].

Our study has some limitations. First, this is a retrospective analysis, and access to some necessary specific details was limited; this resulted in the exclusion of several cases. Second, the analyzed data came from a single medical center. Multicenter analysis would be more reliable for analysis. Third, although we confirmed that CSA may result in overdiagnosis and overtreatment, we have provided only indirect evidence of this phenomenon. However, as we stated previously, it is very difficult to confirm our observations unless some patients who do not undergo surgery are followed until they die of other causes.

## Conclusions

CSA discovers indolent cases of PTC and might be one of the causes of the overdiagnosis and overtreatment of this malignancy. However, we revealed only indirect evidence of this phenomenon. To identify patients with PTC who are diagnosed at an early stage but are not overdiagnosed and overtreated, some risk-based screening tools are needed. In this specific cancer type, new approaches should be introduced.

## Supporting information

S1 Data (XLS)
